# Defining a glycemic persistence index (GPI) for continuous glucose monitoring

**DOI:** 10.21203/rs.3.rs-8841862/v2

**Published:** 2026-03-19

**Authors:** Ren Zhang

**Keywords:** Continuous glucose monitoring, CGM, glycemic persistence index, GPI, hyperglycemia

## Abstract

Sustained hyperglycemia is the core driver of diabetes complications. Continuous glucose monitoring (CGM) provides rich temporal glucose data, but effective summarization that integrates both magnitude and duration of sustained hyperglycemia into a single and paramter-free scalar remains challenging. We introduce the glycemic persistence index (GPI), a simple, threshold-free CGM-derived metric defined as the largest integer k such that at least k minutes are spent at glucose levels ≥ k mg/dL within a day. For example, a GPI of 125 means that glucose levels were ≥ 125 mg/dL for at least 125 minutes during that day. Geometrically, after ranking glucose values in decreasing order, GPI is given by the intersection at which glucose level and cumulative duration take the same value. Using CGM data from the International Diabetes Closed Loop randomized trial (168 adults with type 1 diabetes), GPI sensitively captured closed-loop treatment benefit with significantly lower values in the closed-loop group compared with sensor-augmented pump therapy (P = 0.0001). GPI correlated with HbA1c and daily mean glucose, while distinguishing patterns of sustained hyperglycemia among individuals with similar conventional metrics. As a simple, device-independent, and threshold-free scalar, GPI quantifies hyperglycemia by jointly capturing its magnitude and duration, enabling consistent and intuitive glycemic profiling accessible to both specialists and non-specialists.

## Introduction

Diabetes and dysglycemia are highly prevalent worldwide, spanning a continuum from prediabetes to established diabetes and affecting an increasing proportion of adults^1,2^. Across this spectrum, chronic exposure to elevated glucose is a central determinant of metabolic burden and long-term risk^3,4^.

Continuous glucose monitoring (CGM) has transformed glycemic assessment by providing high-frequency measurements in daily life. These data capture rich temporal structure, including fluctuations, excursions, and prolonged periods of dysglycemia, but their volume and complexity necessitate effective summarization into concise, interpretable metrics. In both clinical practice and randomized trials, CGM-derived summaries are routinely used to evaluate glycemic control and therapeutic efficacy^5,6^. Commonly reported metrics include daily mean glucose, measures of glycemic variability, and threshold-based summaries such as time-in-range (TIR) ^7–11^. For example, TIR served as the primary efficacy endpoint in the International Diabetes Closed Loop (DCLP3) randomized trial, in which closed-loop therapy significantly improved TIR compared with control^12^.

Threshold-based metrics, however, require prespecified glucose cutoffs. These cut points may differ across populations, including type 1 diabetes, type 2 diabetes, and prediabetes, and results can vary depending on the selected thresholds. Once defined, such metrics provide no further gradation above the chosen boundary. Moreover, sustained hyperglycemia reflects not only how long glucose remains elevated but also how high it rises. Mean glucose summarizes overall exposure, and variability measures describe dispersion, but neither directly integrates magnitude and duration into a unified representation of persistence. A metric that captures glycemic persistence should therefore encode both dimensions within a single, interpretable scalar while remaining independent of arbitrary cutoffs.

To address these challenges, we introduce the glycemic persistence index (GPI), a simple, nonparametric metric derived from CGM data that integrates glucose magnitude and duration into a single scalar expressed in minutes. GPI is immediately interpretable and does not rely on predefined glucose thresholds or modeling assumptions. Using CGM data from the DCLP3 randomized trial, we evaluate GPI and examine its relationship to existing CGM summaries.

## Materials and methods

### Continuous glucose monitoring data

CGM data were derived from the International Diabetes Closed Loop (iDCL) randomized trial, which enrolled 168 participants with type 1 diabetes (112 assigned to closed-loop therapy and 56 to sensor- augmented pump therapy)^12^. The study duration was 26 weeks. Glucose measurements were obtained using the Dexcom G6 continuous glucose monitoring system (Dexcom), which records interstitial glucose values every 5 minutes (up to 288 measurements per day). Raw timestamped CGM data were analyzed without smoothing or interpolation. Timestamps were converted from SAS datetime format to standard datetime objects. For each participant, glucose values were aligned relative to the randomization date. Baseline CGM data were defined as recordings obtained prior to randomization during the run-in period. Glucose measurements were grouped by participant and calendar date to generate daily glucose profiles. Days with fewer than 200 valid CGM readings were excluded to ensure adequate data completeness. All analyses were conducted at the subject-day level.

### Definition of the glycemic persistence index (GPI)

For each subject-day, CGM measurements were used to compute the GPI. Glucose values within a day were represented as a time series with corresponding timestamps and ranked in decreasing order of glucose concentration. Cumulative time was computed based on the native sampling interval, yielding the total duration (in minutes) associated with glucose values exceeding a given level.

Let $G_{t}$ denote the glucose concentration (mg/dL) measured at time t, and let $\text{Δ}t_{t}$ denote the duration (in minutes) represented by that CGM measurement, determined by the native sampling interval. The GPI was defined as

$$\text{GPI}=\max\left\{k\in \mathbb{N}:\sum_{\left\{t:G_{t}\setminus \text{gek}\right\}}\text{Δ}t_{t}\setminus \text{gek}\right\}.$$

This definition identifies the largest integer value k for which the cumulative time spent at glucose values greater than or equal to k mg/dL is at least k minutes. Thus, GPI jointly encodes glucose magnitude and duration on a common numerical scale, increasing when higher glucose levels persist for longer periods. GPI can be computed by iterating over integer glucose levels k, summing CGM durations at or above k, and identifying the maximum k satisfying the persistence condition. GPI is expressed in units of time (minutes) and is evaluated over integer values of k, ensuring that it is independent of CGM sampling frequency and directly comparable across devices and study settings.

### Statistical analysis, software, and visualization

Daily GPI values were computed for each subject-day using quality-controlled CGM data. For longitudinal treatment analyses, daily GPI values were averaged within each participant during the baseline period and during the post-randomization period (weeks 1–26). Between-group comparisons of post- randomization GPI were performed using analysis of covariance (ANCOVA), with treatment group included as a fixed effect and baseline mean GPI as a covariate. For subject-day-level analyses, daily mean glucose and daily glucose variance were calculated using all available CGM measurements within each 24-hour period. Associations between daily GPI and conventional glycemic metrics were assessed using Pearson correlation coefficients. All statistical tests were two-sided, and *P* values < 0.05 were considered statistically significant. Data processing and statistical analyses were performed using Python 3 (statsmodels). Data manipulation was conducted using standard scientific computing libraries, and figures were generated using matplotlib.

## Results

### Geometric definition of the glycemic persistence index

**Figure 1:**
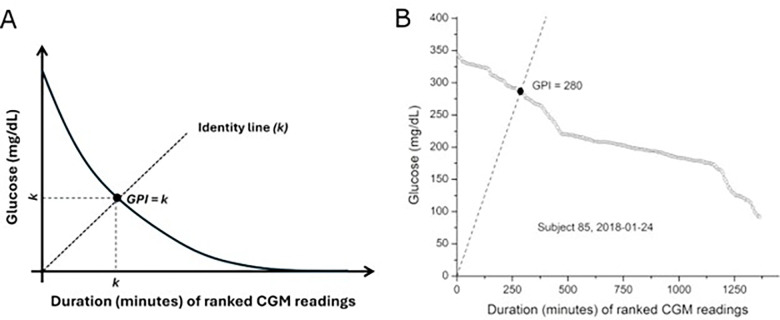
Geometric definition of the glycemic persistence index (GPI). A) Glucose measurements from a single day are ranked in descending order and plotted against time (minutes). The dashed identity line (k) represents the index-defining condition in which the same scalar k is applied to both duration and glucose magnitude. The GPI is defined as the largest value k for which at least k minutes exhibit glucose concentrations ≥ k mg/dL, corresponding to the intersection of the ranked glucose curve with the identity line. B) Representative CGM example. Ranked 24-hour CGM glucose values (5-minute sampling) from a representative participant (Subject 85, January 24, 2018). A GPI of 280 indicates sustained hyperglycemia ≥280 mg/dL for at least 280 minutes.

Geometrically, GPI is defined from ranked daily CGM data (Fig. 1A). For each subject-day, glucose measurements were ordered in descending magnitude and plotted against cumulative duration, yielding a monotonic decreasing curve that jointly encodes glucose intensity and exposure time. GPI is the maximal value k such that glucose levels ≥ k mg/dL are sustained for ≥ k minutes within a 24-hour period. This value corresponds to the intersection between the ranked glucose curve and the identity line (y = x), where glucose magnitude and duration are expressed on a common numerical scale. The horizontal and vertical projections of this intersection define the shared scalar k (Fig. 1A). By jointly encoding severity and persistence in a unified geometric framework, GPI provides an interpretable summary of daily hyperglycemic burden. Larger GPI values reflect days characterized by sustained exposure to elevated glucose, whereas lower values indicate either shorter durations or lower magnitudes of hyperglycemia. For example, GPI for subject 85 on January 24, 2018, was 280, indicating that the subject spent at least 280 minutes with glucose levels ≥ 280 mg/dL during the 24-hour period (Fig. 1B). This geometric construction yields a single scalar measure that integrates intensity and persistence of hyperglycemia.

### GPI reflects treatment effects during randomized closed-loop therapy

The International Diabetes Closed Loop (iDCL) study was a 26-week, multicenter, randomized controlled trial involving 168 participants with type 1 diabetes assigned in a 2:1 ratio to closed-loop insulin delivery or sensor-augmented pump therapy. In that trial, the primary efficacy endpoint was the percentage of time that glucose levels were within the target range of 70–180 mg/dL (time-in-range, TIR), with closed-loop therapy demonstrating a significant improvement in TIR ^12^. To determine whether GPI reflects clinically relevant treatment effects in a randomized controlled setting, we analyzed GPI using the same CGM dataset from the iDCL study. Specifically, we examined (1) whether GPI differs between treatment groups over 26 weeks of follow-up and (2) how GPI relates to TIR, the prespecified primary endpoint of the parent trial.

**Figure 2:**
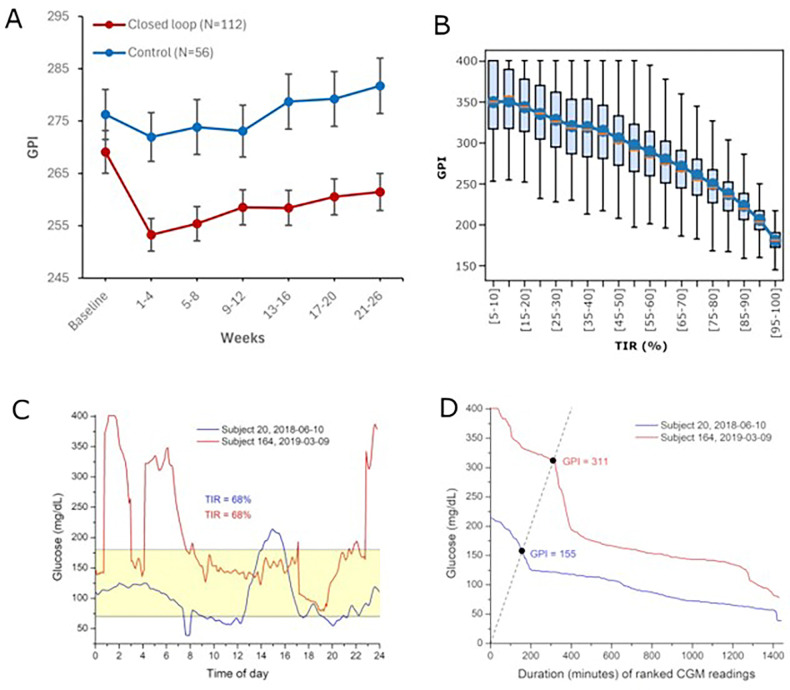
Glycemic persistence index (GPI) captures treatment effects and sustained hyperglycemia. A) Longitudinal changes in daily GPI over 26 weeks of treatment. Mean GPI at baseline and across 4-week intervals through 26 weeks in the closed-loop group (red; N = 112) and sensor-augmented pump (control) group (blue; N = 56). Points show subject-weighted means; error bars indicate standard errors. Lower GPI reflects reduced sustained hyperglycemia. During 26 weeks, mean daily GPI was significantly lower in the closed-loop group after adjustment for baseline GPI (*P* = 0.0001, ANCOVA). B) GPI According to TIR Bands. Daily GPI values during weeks 1–26 are shown within 5–percentage-point categories of TIR (70–180 mg/dL). Boxes represent interquartile ranges, horizontal lines medians, whiskers full ranges, and circles means. Although GPI inversely correlates with TIR, substantial variability within TIR bands indicates that glycemic persistence is not fully captured by TIR. C) CGM traces from two subjects with comparable TIR (70–180 mg/dL) but marked differences in glycemic persistence. (D) Higher GPI reflects more sustained hyperglycemia in the same subjects.

At baseline, mean daily GPI values were similar between the closed-loop and control groups. After randomization, a consistent separation between groups became evident. In participants assigned to closed-loop therapy, mean GPI declined during the first post-randomization interval and remained lower than baseline across all subsequent 4-week periods through week 26. In contrast, the control group showed minimal change over time, with mean GPI remaining near baseline levels (Fig. 2A). The between- group difference was maintained across the entire 26-week period without attenuation. Variability, reflected by standard error bars, remained relatively stable in both groups over time, suggesting that the observed difference was not driven by increasing dispersion. After adjustment for baseline GPI, mean post-randomization GPI was significantly lower in the closed-loop group than in the control group (*P* = 0.0001). Because higher GPI values indicate longer duration at higher glucose levels, the reduction observed in the closed-loop group reflects decreased persistence of hyperglycemia during treatment.

Given that TIR was the primary endpoint of the parent randomized trial, we examined daily GPI values stratified by 5–percentage-point TIR categories during weeks 1–26. Median GPI decreased progressively across higher TIR strata, consistent with an inverse association between time spent within 70–180 mg/dL and glycemic persistence. However, substantial dispersion in GPI was observed within individual TIR categories. Even among days with similar TIR percentages, GPI values spanned a broad range, indicating heterogeneity in the magnitude and duration of hyperglycemia not fully characterized by TIR alone. To illustrate this distinction, CGM traces from two participants with identical TIR values (68%) but markedly different GPI values are shown (Fig. 2C–D). Subject 164 exhibited prolonged hyperglycemia with sustained glucose levels exceeding 300 mg/dL, resulting in a GPI of 311. In contrast, Subject 20 had shorter and lower-amplitude excursions, with glucose rarely exceeding approximately 220 mg/dL, yielding a GPI of 155. Despite identical TIR, the 156-point difference in GPI (311 vs. 155) reflects substantially greater persistence of severe hyperglycemia in Subject 164, corresponding to 156 additional minutes during which very high glucose levels were sustained.

### GPI in relation to HbA1c and daily mean glucose

**Figure 3:**
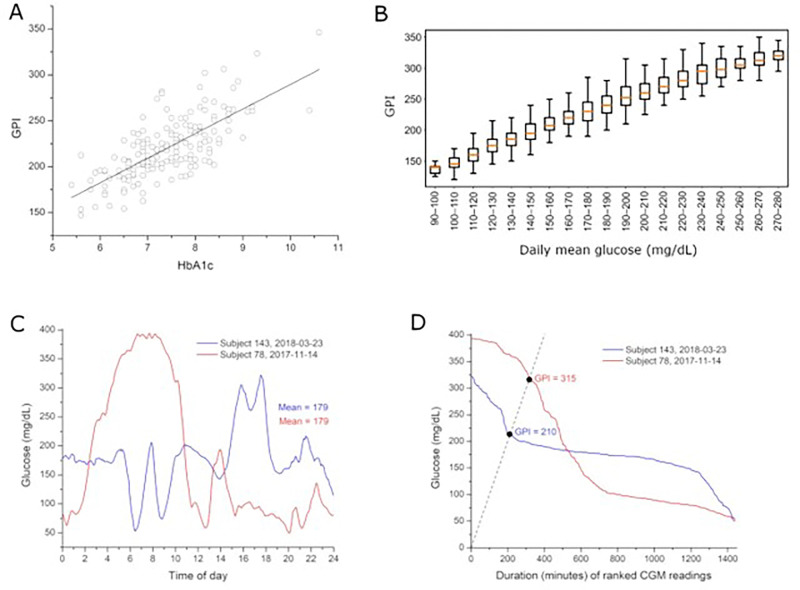
Relationship between baseline GPI, HbA1c, and mean glucose. A) Baseline GPI correlated with HbA1c (n = 168; Pearson’s *r* = 0.72, *P*< 0.001), yet substantial variability in GPI at comparable HbA1c levels indicates that hyperglycemia persistence is not fully captured by HbA1c. B) Baseline CGM data were analyzed at the subject-day level (n = 2,162 baseline days from 168 participants). Daily mean glucose values were grouped into 10 mg/dL bins. Within each bin, box plots display the distribution of daily GPI values (median, interquartile range, and whiskers representing the full range excluding outliers). Although GPI increases progressively with higher mean glucose, substantial dispersion in GPI is observed within individual mean glucose strata. (C–D) Representative baseline subject-days with identical mean glucose but different GPI values. Despite the same average glucose (179 mg/dL), Subject 78 (GPI = 315) exhibited substantially greater persistence of hyperglycemia than Subject 143 (GPI = 210).

To examine how GPI relates to established glycemic metrics, we analyzed baseline GPI in relation to HbA1c and daily mean glucose. Baseline GPI was positively correlated with baseline HbA1c across 168 participants (Pearson’s *r* = 0.72, *P* < 0.001; Fig. 3A). Higher HbA1c values were generally associated with higher GPI values. However, substantial dispersion in GPI was observed at comparable HbA1c levels, indicating heterogeneity in glycemic persistence among individuals with similar chronic glycemic exposure. At the subject-day level (n = 2,162 baseline days), daily mean glucose was grouped into 10 mg/dL categories. Median GPI increased progressively across higher mean glucose strata (Fig. 3B), consistent with greater persistence of hyperglycemia at higher average glucose levels. Despite this monotonic trend, wide within-bin variability in GPI was observed across all mean glucose categories. Even among days with similar mean glucose values, GPI values spanned broad ranges, indicating that mean glucose does not uniquely determine the magnitude–duration structure of hyperglycemia. To further illustrate this distinction, we examined two representative baseline subject-days with identical mean glucose (179 mg/dL) but differing GPI values (Fig. 3C–D). Subject 78 exhibited sustained high- amplitude hyperglycemia early in the day, resulting in a GPI of 315. In contrast, Subject 143 demonstrated shorter and lower-magnitude excursions, yielding a GPI of 210 despite the same daily mean glucose. The 105-point difference in GPI reflects 105 additional minutes of sustained severe hyperglycemia in Subject 78. These examples suggest that GPI differentiates glycemic patterns that are not fully characterized by HbA1c or daily mean glucose alone.

## Discussion

In this study, we introduce GPI as a simple, nonparametric CGM-derived metric that integrates glucose magnitude and duration into a single scalar expressed in minutes. In a randomized clinical trial dataset, GPI was responsive to treatment and correlated strongly with mean glucose and glycemic variability, yet remained non-redundant with either measure, indicating that it captures an additional dimension of glycemic burden. Conceptually, GPI reflects the persistence of elevated glucose by jointly encoding how high glucose rises and how long it remains elevated. By emphasizing sustained hyperglycemia rather than brief excursions or overall dispersion, GPI provides a concise representation of prolonged glycemic exposure.

The observed relationships between GPI and established CGM summaries help position it within the existing metric framework. Mean glucose reflects overall exposure, variability measures describe dispersion, and threshold-based metrics quantify time within or outside prespecified ranges. In contrast, GPI characterizes the magnitude–duration structure of elevated glucose. By jointly encoding how high glucose rises and how long it remains elevated, GPI captures sustained hyperglycemic burden in a way that is mathematically distinct from both average exposure and dispersion-based measures. Accordingly, GPI is considered complementary to existing summaries.

GPI offers several practical advantages. 1) GPI does not rely on predefined glucose cutoffs. In clinical practice and trials, metrics such as TIR or TAR require prespecified thresholds, which may differ across populations and study designs, potentially yielding different results depending on the selected cut points. Once a threshold is crossed, such metrics provide no further gradation of severity. In contrast, GPI requires no externally imposed ranges and adapts continuously to the observed glucose profile, thereby avoiding sensitivity to chosen cutoffs. 2) GPI also condenses two-dimensional information, magnitude and duration, into a single scalar. Metrics such as mean glucose or standard deviation capture only one dimension of glycemia, whereas GPI jointly encodes how high glucose rises and how long it remains elevated. This integration allows sustained hyperglycemia to be summarized in a concise and unified manner. 3) GPI is computationally simple and assumption-free. It can be derived directly from CGM data without modeling, smoothing, or distributional assumptions and is directly comparable across devices and study settings. 4) An important advantage is its intuitive interpretability. Because GPI is expressed in minutes, its meaning is immediate: for example, GPI = 120 indicates that at least 120 minutes were spent at glucose levels ≥ 120 mg/dL. This direct magnitude–duration correspondence facilitates interpretation by both specialists and non-specialists.

Several limitations should be acknowledged. Analyses were based on CGM data from a single randomized clinical trial, and associations with long-term clinical outcomes were not evaluated. Accordingly, the present study focuses on defining and characterizing GPI rather than establishing its prognostic or therapeutic implications. Future studies should assess GPI in broader and more diverse populations, including different diabetes subtypes and varying degrees of dysglycemia, and examine its relationship to complications, treatment response, and longitudinal risk markers. Integration of GPI into standardized CGM reporting frameworks may further clarify its potential role in clinical assessment.

In summary, GPI quantifies glycemic persistence by integrating glucose magnitude and duration into a single scalar expressed. It is threshold-free, device-independent, and can be derived directly from CGM data without modeling assumptions. Because its value corresponds directly to matched units of glucose and time, GPI is immediately interpretable, providing a concise and intuitive representation of sustained hyperglycemic burden.

## Data Availability

The CGM dataset analyzed in this study is publicly available from the International Diabetes Closed Loop (DCLP3) trial repository. The Python scripts used for analysis are available from the corresponding author upon reasonable request.
